# A Simple Method of Determining the Blood Coagulation Time

**Published:** 1907-12-14

**Authors:** 


### Clinical Points.

### A SIMPLE METHOD OF DETERMINING THE BLOOD COAGULATION-TIME.

A considerable amount of attention is being devoted nowadays to the coagulability of the blood in different conditions, more especially in cases of jaundice, of spontaneous hæmorrhage, of venous thrombosis, and the like. It is well known, of course, that calcium is an essential factor in the clotting of blood; but it is also well known that, whereas a certain proportion of calcium assists coagulation, a point is presently reached at which the presence of more calcium actually delays the clotting. It is, therefore, possible to do harm instead of good by administering calcium salts in too large a quantity.

The difficulty has hitherto been that the instruments devised for measuring the coagulability of the blood have been for the most part not only expensive, but also complicated. The best-known coagulometer in this country is probably the one in which blood is drawn up into a series of capillary tubes which are kept standing in a water-bath at body temperature. The observer blows into the successive capillary tubes at intervals, until he comes to one from which the blood cannot be readily expelled; this is the one in which clotting has just taken place, and the time since it was first filled with blood is noted. This is a fairly easy procedure so far, but now follows the necessity of making all the capillary tubes absolutely clean again, in readiness for a subsequent determination. The cleaning of those tubes in which no clotting has occurred is not so difficult, though it takes time to clean a dozen; the cleaning of a capillary tube in which clotting has taken place is a much longer and a much more irritating process. It is small wonder therefore that this coagulometer has not been widely used in general practice.

A large number of other methods of measuring the coagulation-time of the blood have been devised. We do not propose to discuss them all; we will rather restrict ourselves to that which is the simplest—namely,

### Milian's Slide Method, as modified by Hinman and Sladen. The only apparatus required is a microscope slide with a millimetre scale. The latter may be scratched upon the glass itself; or a piece of paper may be marked off in millimetres, and gummed on to the microscope slide with the marked surface of the scale next to the glass. The microscope slide can then be laid flat upon a table, with its clean glass surface upwards; the paper millimetre scale on its under surface can be seen through the glass. The object of the scale is simply to be sure of the size of the blood-drop that is being taken. It takes longer for a big drop to become completely clotted than it does for a smaller drop, so that it is necessary, for purposes of comparing one coagulation-time with another, that the blood-drops in successive determinations should be approximately the same size. The actual diameter of the drops is immaterial, provided that it is always about the same, and that the normal coagulation-times have been already determined for a blood-drop of the diameter selected.

The coagulation-times for blood-drops four millimetres in diameter have been fully determined; it is therefore convenient to fix the size at four millimetres in practice.

The lobule of the ear is punctured in the usual way, the first blood-drop is wiped away, and the time is counted from the appearance of the second drop. The diameter of the drop picked off depends mainly upon the size of the globule on the ear. It is of advantage to hold the lobule of the ear out, so that the slide may be horizontal when touched to the drop, and to touch the drop with the under surface of the slide rather than to receive the drop on its upper surface. The slide is then quickly turned to prevent the blood from flowing about. A series of drops may be taken upon the same slide. One of such a series is sure to cover approximately four millimetres of the scale, and this one should be retained, the rest being wiped away. It is well to prepare more than one slide with a suitable drop on it in this way, timing each separately, so that if any accident or doubt arises in connection with the first, the second can be used in its stead.

There are two ways of watching these drops to determine when coagulation has occurred. The first is to observe its contour from the side; before coagulation the contour of the blood-drop is smooth and rounded, whilst when coagulation has taken place it becomes flattened and irregular. The second is to hold the slide to transmitted light and judge if there is a flow in the drop by the changes in density. The denser portion is in the centre of the drop when coagulation has occurred, whereas it is at the lower edge of the drop, whichever way the slide is tilted, when the drop is still fluid. It is well to combine these two methods as an aid in judgment of the end point. As a rule it is easy to determine when coagulation has progressed far enough to prevent a change in the contour on tilting the slide. Each observation may be confirmed by picking up a macroscopic clot with the tip of a cloth or filter-paper.

### The Normal Blood Coagulation-time.

It is not possible to say absolutely "the blood coagulation-time in this patient is so-and-so"; one can only say that "the blood coagulation-time in this patient *as estimated by such and such a method* is so-and-so." In giving the coagulation-time the method used should be stated; the results obtained by one method are not comparable with those obtained by another method. It is important that, in the simple slide method, the drops should be always about the same size, and that they should not be exposed to undue drying influences, such as the heat of the sun or a fire, or a current of air.

It will then be found that, with a four-millimetre drop, the coagulation-times for normal blood vary between five and eight minutes. In some diseases in which it might be thought that clotting power was deficient, it is found to be normal; in others it is notably delayed; in some cases of the latter the effects of calcium have been negative, in others markedly beneficial.

